# Pangenomic framework for globe artichoke supports SNP‐based varietal fingerprinting

**DOI:** 10.1002/tpg2.70333

**Published:** 2026-10-22

**Authors:** Ezio Portis, Edoardo Vergnano, Luciana Gaccione, Alberto Acquadro, Cinzia Comino, Cristiano Carli, Lorenzo Barchi, Matteo Martina

**Affiliations:** ^1^ Dipartimento di Scienze Agrarie, Forestali e Alimentari (DISAFA), Plant Genetics Università degli Studi di Torino Torino Italy; ^2^ AGRION Fondazione per la ricerca, l'innovazione e lo sviluppo tecnologico dell'agricoltura piemontese Cuneo Italy

## Abstract

Globe artichoke (*Cynara cardunculus* var. *scolymus* L.) comprises a broad range of local ecotypes and varietal groups whose genetic diversity has been investigated through different molecular markers. However, recent advances in next‐generation sequencing and pangenomic approaches provide new opportunities to capture genome‐wide variation at higher resolution and to develop practical tools for varietal discrimination, traceability, and germplasm conservation. In this study, we developed the first pangenomic framework for cultivated artichoke and evaluated pangenome‐informed single‐nucleotide polymorphism (SNP) markers for varietal fingerprinting. Whole‐genome resequencing data from the Italian local ecotype Asti Sorì were integrated with publicly available genomic data from representative globe artichoke and cultivated cardoon accessions to construct and annotate a pangenome. Genome‐wide SNP and presence/absence variation (PAV) analyses were combined with pangenome‐anchored genotyping‐by‐sequencing (GBS) data from 45 accessions representing the main cultivated varietal groups. The pangenome revealed a largely conserved core gene repertoire alongside a smaller accessory component, with gene accumulation curves suggesting a tendency toward saturation within the sampled cultivated germplasm. SNP‐ and PAV‐based analyses provided complementary views of accession relationships and consistently resolved the principal cultivated groups. Across the broader germplasm panel, pangenome‐anchored GBS‐derived SNPs identified well‐supported phylogenetic clusters corresponding to recognized varietal types. The best‐performing 50‐SNP panel showed a high correlation with the pairwise genetic‐distance matrix obtained from the complete dataset (Pearson's *r* = 0.96) and largely reproduced its main varietal structure. This compact marker set provides a practical foundation for varietal fingerprinting, DUS (distinctness, uniformity, and stability)‐oriented applications, traceability, and conservation of traditional globe artichoke germplasm.

## Plain Language Summary

Globe artichoke includes many traditional local varieties that are important for regional agriculture, conservation, and breeding. Their genetic diversity has been investigated in previous studies, but recent advances in next‐generation sequencing and pangenomics offer new opportunities to describe this diversity with higher resolution. We developed a pangenomic resource that better represents the diversity present within cultivated material. Using the Italian local ecotype Asti Sorì as a case study, we compared its genome with those of other globe artichoke and cultivated cardoon accessions. We identified shared and accession‐specific genetic features, showing that varietal groups can be distinguished using DNA markers. We also developed a reduced set of 50 genetic markers that reproduced the main diversity patterns observed in a broader collection. These markers could support varietal identification, traceability, conservation of traditional germplasm, and future breeding efforts.

**Abbreviations**
BH: Benjamini–Hochberg
CTAB: cetyltrimethylammonium bromide
DUS: distinctness, uniformity, and stability
GBS: genotyping‐by‐sequencing
GO: Gene Ontology
PAV: presence/absence variation
PCA: principal component analysis
RADseq: restriction site‐associated DNA sequencing
SNP: single‐nucleotide polymorphism
SRA: Sequence Read Archive
SV: structural variant

## 1 INTRODUCTION

Pangenome approaches are reshaping how intraspecific genomic diversity is studied in crop species. By integrating sequence data from multiple accessions, pangenomes provide access to genomic content not represented in any single reference assembly—including structural variants (SVs), presence/absence variation (PAV), and accession‐specific sequences (Bayer et al., 2020; Golicz et al., 2016; Schreiber et al., 2024). In crops characterized by complex domestication histories, clonal propagation, or pronounced agro‐ecological differentiation, single‐genome references are increasingly recognized as inadequate representations of species‐wide diversity (Chapman et al., 2022; Schreiber et al., 2024). Pangenomic frameworks have been applied to improve marker discovery, characterize population structure, and identify loci associated with traits of agronomic importance across a range of vegetable and fruit crops (N. Li et al., 2023; Liu et al., 2023; Zhou et al., 2022).

Globe artichoke (*Cynara cardunculus* var. *scolymus* L.) is a diploid, cross‐pollinating member of the Asteraceae family, primarily cultivated across the Mediterranean basin, with an estimated genome size of approximately 1.084 Gb (Acquadro et al., 2020; Scaglione et al., 2016). Morphological and molecular studies have identified differentiated varietal groups, including Catanese/Violetto, Spinoso, Romanesco, and Violetto di Toscana types (Martina et al., 2024; Miccolis & Elia, 1996; Pavan et al., 2018; Portis et al., 2025; Rau et al., 2022). However, the genomic basis of this structure and the extent of non‐reference sequence variation remain poorly characterized.

Local ecotypes occupy a distinct segment of crop diversity: maintained by farmers over generations through empirical selection in specific agro‐ecological contexts, they often preserve allele combinations that are not present in improved or commercial varieties (Cheng et al., 2024). The globe artichoke ecotype Asti Sorì has been cultivated in the Asti area of Piedmont (northwestern Italy) for local fresh markets and is recognized by local producers for its distinctive agronomic characteristics. Despite its historical presence in the territory, Asti Sorì has not been characterized at the genomic level, and no molecular tools are available for its formal identification or protection.

No pangenomic resource currently exists for globe artichoke, limiting the completeness of diversity analyses and the characterization of non‐reference genomic content. At the same time, the absence of standardized single‐nucleotide polymorphism (SNP)‐based marker panels constrains varietal registration, DUS (distinctness, uniformity, and stability) testing, and the molecular traceability of traditional ecotypes. Previous genotyping‐by‐sequencing (GBS) studies based on RADseq (restriction site‐associated DNA sequencing)‐derived markers demonstrated the potential of genome‐wide SNP discovery for the analysis of globe artichoke population structure and diversity (Acquadro et al., 2015; Comino et al., 2016), but these approaches were not anchored to a pangenomic framework and were not specifically optimized for reduced diagnostic marker panel development. Accession‐specific variants identified from limited resequencing panels may provide an initial route for marker discovery; however, loci appearing private within small discovery sets may not retain discriminatory power when evaluated across broader germplasm collections. A population‐level approach based on pangenome‐anchored GBS‐derived SNPs therefore provides a more scalable route toward practical varietal fingerprinting tools.

To provide an initial but structured representation of the available cultivated diversity, we selected six accessions spanning major varietal backgrounds: A41 (cultivated cardoon), C3 (Romanesco), SP (Spinoso), VS (Violetto di Sicilia), VT (Violetto di Toscana), and AS (Asti Sorì). This panel is not intended to capture the full species‐wide diversity, but to provide a first comparative framework including the local ecotype under study, major cultivated groups, and one related cultivated cardoon.

In this study, we pursued three aims: (i) to generate whole‐genome resequencing data for Asti Sorì and integrate them with publicly available genome data from representative *C. cardunculus* accessions and the 2C reference genome; (ii) to reconstruct and annotate accession‐level genomes and quantify pangenome content, including SNP and PAV; and (iii) to evaluate pangenome‐anchored GBS‐derived SNPs in a broader germplasm panel and derive a preliminary reduced marker set for varietal fingerprinting.

## 2 MATERIALS AND METHODS

### 2.1 Plant material and agronomic evaluation

The local globe artichoke ecotype Asti Sorì was evaluated under field conditions in two distinct environments in the Province of Asti (Piedmont, northwestern Italy), characterized by different pedoclimatic conditions and cultivation practices. Experimental trials were conducted in the municipalities of Costigliole d'Asti and Revigliasco, including approximately 600 and 400 clonally propagated plants, respectively. At each site, ten representative plants were selected for detailed phenotypic assessment. Standard local agronomic practices were applied, including seasonal sucker removal to regulate vegetative growth and maintain plant productivity. Phenotypic observations included plant vigor, head number per plant, head weight, and head morphology (diameter, height, and shape), together with general observations on phenological development and plant health status throughout the growing season. The agronomic evaluation was performed to document the phenotypic consistency of the local material across environments and to support the genomic characterization of the Asti Sorì ecotype used in subsequent analyses. To assess genetic variation within varietal types, a total of 45 globe artichoke accessions derived from a broader globe artichoke germplasm collection representative of the major cultivated varietal groups (Comino et al., 2016; Rau et al., 2022) were selected for DNA extraction and subsequent molecular analyses. Globe artichoke accessions were subdivided into seven groups based on morpho‐phenotypic traits, primarily related to head characteristics (Table S1).

### 2.2 DNA extraction, sequencing, and data collection

Genomic DNA was extracted from fresh/lyophilized young leaves of each accession using a modified cetyltrimethylammonium bromide protocol (Doyle, 1991; R. Li et al., 2024). RNAse A was used to remove RNA contamination, and DNA quality was checked by 1% (w/v) agarose gel electrophoresis. Quantification was performed by Qubit 2.0 (Life Technologies), based on the Qubit dsDNA BR Assay (Life Science), following Martina et al., 2023. Whole‐genome sequencing of Asti Sorì was performed at approximately 35× coverage using an Illumina NovaSeq X platform with 150‐bp paired‐end reads. In addition, publicly available resequencing data from representative Italian globe artichoke varietal types Violetto di Sicilia (VS), Violetto di Toscana (VT), Romanesco C3 (C3), Spinoso di Palermo (SP), and the cultivated cardoon Altilis 41 (A41) were retrieved from the NCBI Sequence Read Archive (SRA—PRJNA238069; Acquadro et al., 2017). These materials included five cultivated globe artichoke accessions (four from PRJNA238069 plus Asti Sorì) and one cultivated cardoon accession, and were used for genome reconstruction, comparative analyses, and pangenome construction.

### 2.3 Reference‐guided genome reconstruction and annotation

Genome reconstruction of the selected varietal types was performed using a reference‐guided approach. Cleaned reads from each accession were assembled independently into initial contigs using MEGAHIT, an ultra‐fast and memory‐efficient NGS assembler optimized for metagenomic datasets but also applicable to generic single‐genome assemblies (D. Li et al., 2015). The resulting contigs were subsequently scaffolded against the globe artichoke reference genome (V2; Acquadro et al., 2020) using RagTag (Alonge et al., 2022). Contigs shorter than 500 bp were discarded. Structural gene annotation was carried out using a combined approach integrating ab initio prediction and transcript evidence. Gene models were predicted using Helixer and BRAKER3 (Gabriel et al., in press; Holst et al., 2026; Stiehler et al., 2021), incorporating publicly available RNA‐seq data from the *C. cardunculus* transcriptome project (NCBI BioProject PRJNA72327; SRA study SRP010589; runs SRR403068, SRR403069, SRR403070, SRR403073, and SRR403074), originally reported by Scaglione et al. (2012). Functional annotation was performed using DIAMOND (v2.1.8.162; Buchfink et al., 2021) against two curated/reference protein databases: UniProtKB/Swiss‐Prot reviewed proteins restricted to Viridiplantae (local database ID: uniprotkb_reviewed_true_AND_taxonomy_id_2025_04_01.dmnd; snapshot: April 1, 2025; 41,876 sequences; https://www.uniprot.org/; Bateman et al., 2023) and NCBI RefSeq plant proteins restricted to Viridiplantae (local database ID: refseq_viridi; snapshot: July 19, 2024; 6,806,076 sequences; https://www.ncbi.nlm.nih.gov/refseq/; O'Leary et al., 2016).

### 2.4 Pangenome construction

A pangenome was constructed by integrating the reconstructed genomes of all accessions with the reference genome. The pangenome comprised seven genome sequences: the 2C reference genome and six accession‐level reconstructions, namely A41, AS, C3, SP, VS, and VT. These accessions were selected to represent the major varietal backgrounds available in the dataset, including Romanesco, Spinoso, Violetto, Violetto di Toscana, the local ecotype Asti Sorì, and one cultivated cardoon. This panel was intended as an initial comparative framework rather than a complete representation of the species‐wide diversity. De novo assemblies from all reconstructed accessions were aligned to the reference using minimap2 (H. Li, 2021), and non‐aligned sequences and unaligned regions longer than 500 bp were extracted as candidate non‐reference sequences. Redundant sequences were collapsed using MMseqs2 (Steinegger & Söding, 2017), and contaminant sequences were filtered through similarity searches against the NCBI nonredundant nucleotide (nt) database. The final nonredundant sequences were merged with the reference genome to generate the globe artichoke pangenome. To quantify pangenome expansion, the cumulative number of distinct genes was fitted to Heaps’ law, *P*(*n*) = *κn^γ^*, where *P*(*n*) is the pangenome size after sampling *n* genomes, *κ* is a scaling constant, and *γ* is the expansion exponent. A positive *γ* indicates an open pangenome, whereas *γ* approaching zero indicates a closed pangenome (Tettelin et al., 2005), as implemented in PanGP (Zhao et al., 2014). Genes present in all seven genome sequences were classified as core, genes present in six of seven genomes as softcore, genes present in two to five genomes as shell, and genes present in only one genome as cloud. These frequency‐based thresholds were adopted to distinguish universally conserved, near‐universal, intermediate‐frequency, and rare gene classes.

### 2.5 Functional annotation and Gene Ontology enrichment analysis

Protein sequences were extracted from the pangenome annotation using gffread (v0.12.7; Pertea & Pertea, 2020). Functional annotation was performed using four complementary approaches: (i) eggNOG‐mapper (v2.1.13; Cantalapiedra et al., 2021) with the eggNOG 5.0 database, restricting orthology inference to Viridiplantae (taxid 33090); (ii) InterProScan (v5.67‐99.0; Jones et al., 2014) against all integrated member databases; (iii) DIAMOND (v2.0.15; Buchfink et al., 2021) BLASTp against the UniProt Swiss‐Prot reviewed protein database (downloaded April 2025, e‐value ≤ 1×10^−^^5^); and (iv) DIAMOND BLASTp against the NCBI RefSeq plant protein database (e‐value ≤ 1×10^−^^5^). Functional attributes—including Gene Ontology (GO) terms, Kyoto Encyclopedia of Genes and Genomes pathways, COG categories, and descriptive annotations—were merged by gene identifier into a unified functional table. GO terms were assigned with priority to InterProScan and eggNOG‐derived terms, supplemented by Swiss‐Prot hits. A species‐specific OrgDb annotation package was built using the AnnotationForge package (v1.46.0) in R, incorporating gene‐to‐GO, gene‐to‐pathway, and gene‐to‐symbol mappings for all pangenome genes. GO enrichment analyses were performed in R using the clusterProfiler package (v4.12.0; Yu et al., 2012) with the enrichGO function (Benjamini–Hochberg [BH]‐adjusted *p*‐value cutoff 0.05; *q*‐value cutoff 0.20), using the full set of annotated pangenome genes as the statistical universe. Redundant GO terms were removed using the simplify function (similarity cutoff 0.7). For the Asti Sorì‐unique gene set (*n* = 33), a relaxed threshold (BH *p* < 0.10) was applied given the limited gene number. To assess the functional stratification of the pangenome along the core‐to‐cloud axis, curated macro‐categories of biological function were defined based on six anchor GO term sets (defense and immunity, specialized metabolism, signal transduction, transport, transcriptional regulation, primary metabolism and housekeeping), expanded to all descendant terms using GO ontology offspring relationships. Enrichment of each macro‐category within each pangenome compartment (core, softcore, shell, cloud) relative to the annotated pangenome universe was assessed using Fisher's exact test with BH correction.

### 2.6 Variant calling and population analysis

Sequencing reads from each accession were aligned to the pangenome using BWA‐MEM2 (Vasimuddin et al., 2019). Variant calling was performed using bcftools (Danecek et al., 2021), applying standard quality filters (minimum mapping quality, read depth, and missing data thresholds). Genetic relationships among accessions based on SNP variation were investigated using principal component analysis (PCA) implemented in SNPRelate (Zheng et al., 2012) and maximum‐likelihood phylogenetic inference using IQ‐TREE2 (Minh et al., 2020). PAV analyses were performed using SGSGeneLoss (Golicz et al., 2015), and PAV‐based phylogenetic relationships were compared with SNP‐derived clustering patterns to evaluate complementary patterns of genome diversity across accessions. Candidate SVs were called independently for the six non‐reference accessions using Dysgu (v1.9.0; Cleal & Baird, 2022) in paired‐end mode (*–mode pe*). Because the BAM files had been aligned to the linear pangenome assembly, the same assembly was used as the reference for SV calling to maintain a consistent pangenome coordinate framework. Dysgu was run with *–low‐mem*, *–max‐cov auto*, *–min‐size 50*, *–min‐support 3*, and *–metrics*. For downstream analysis, only variants classified as PASS with at least five supporting evidence units (SU ≥ 5), genotype quality ≥20, and mean primary‐read mapping quality ≥20 were retained. Deletions, insertions, duplications, and inversions were required to have an absolute SVLEN of at least 50 bp, whereas translocations were retained when both breakpoint coordinates were defined. Because the pangenome reference contains sequence derived from the analyzed accessions, these calls quantify read‐supported rearrangements relative to the composite pangenome assembly and should not be interpreted as an unbiased, species‐wide catalogue of structural variation.

### 2.7 GBS‐based genotyping and minimum SNP panel for varietal fingerprinting

To extend comparative analyses to a broader diversity panel, a subset of 45 globe artichoke accessions was selected from a previously characterized germplasm collection representative of the major cultivated varietal groups (Comino et al., 2016; Rau et al., 2022). The selected accessions encompassed the seven principal morpho‐agronomic typologies currently recognized within cultivated globe artichoke germplasm: Catanesi (CAT), Violetti di Provenza (VPR), Violetti di Toscana (VTO), Romaneschi (ROM), Macau (MAC), Spinosi (SPI), and Green et al. (GEA). GBS was performed using a two‐enzyme RADseq approach based on HindIII and NlaIII digestion, with minor modifications from the protocol described by Acquadro et al. (2015). Briefly, genomic DNA was digested with HindIII, ligated to barcoded P1 adapters, pooled, and subsequently digested with NlaIII before ligation to P2 Illumina indexed adapters. Libraries were purified, enriched through PCR to incorporate Illumina sequencing and indexing sites, and sequenced on an Illumina HiSeq 1000 platform according to the manufacturer's protocol.

Sequencing reads were quality‐filtered and aligned to the globe artichoke pangenome generated in this study using BWA‐MEM2 (Vasimuddin et al., 2019). SNP calling was performed using bcftools mpileup/call utilities (Danecek et al., 2021), applying standard quality and depth filters. Pangenome‐anchored SNPs were used to investigate genetic relationships among accessions through PCA and maximum‐likelihood phylogenetic reconstruction using IQ‐TREE2 (Minh et al., 2020). Population stratification within the broader diversity panel was further explored using sparse non‐negative matrix factorization (sNMF) implemented in the LEA package (Frichot & François, 2015), evaluating *K* values from 1 to 20 with 20 independent runs per *K*.

A random SNP subsampling approach was then used to identify reduced marker panels representative of the genetic diversity captured by the full GBS dataset. Starting from the filtered biallelic SNP VCF file, genotype calls were processed in R using the *vcfR* package (Knaus & Grünwald, 2017) and converted into numeric allele dosage values, with homozygous reference, heterozygous, and homozygous alternative genotypes coded as 0, 1, and 2, respectively. Missing genotypes were retained. A reference pairwise genetic distance matrix was first calculated from the complete SNP dataset using dosage‐based Euclidean distances; for each accession pair, distances were computed only on markers with non‐missing genotypes in both accessions and rescaled to account for the number of available markers. For reproducibility, the random‐number generator was initialized with set.seed(123) before random SNP sampling. For each target panel size, corresponding to 50, 75, and 100 SNPs, 100 random SNP subsets were generated without replacement. For each subset, the same distance metric was recalculated and compared with the full‐dataset distance matrix using Pearson correlation across all accession pairs. For each panel size, the subset showing the highest correlation with the full‐dataset distance matrix was retained as the best representative reduced marker panel. The selected SNPs were reported as genomic coordinates and extracted from the original VCF file using *bcftools* (Danecek et al., 2021) for downstream phylogenetic reconstruction and fingerprinting assessment.

## 3 RESULTS

### 3.1 Phenotypic characterization of the Asti Sorì ecotype across two cultivation sites

The local globe artichoke ecotype Asti Sorì is characterized by green, elongated, and spineless capitula, representative of a traditional Northern Italian varietal type (Figure 1).

**FIGURE 1:**
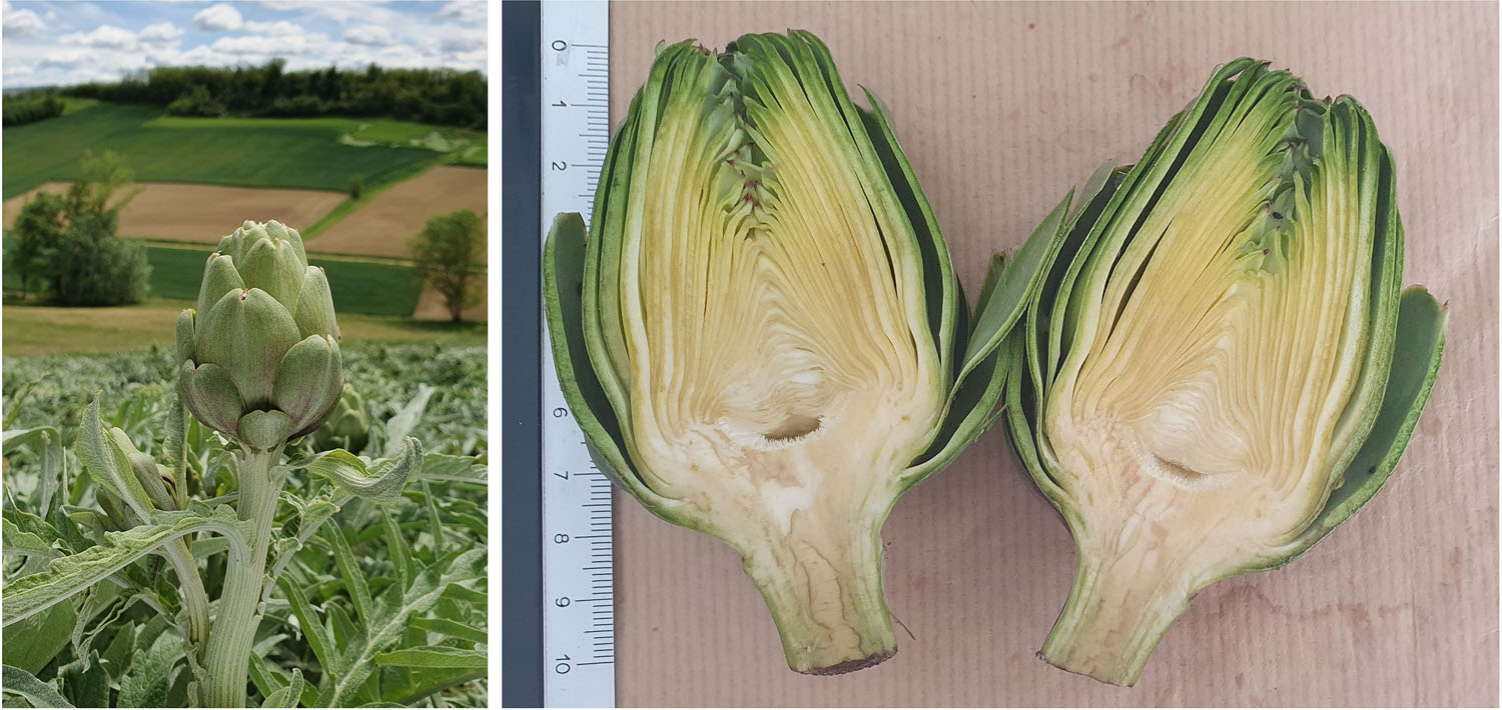
Representative morphology of the globe artichoke ecotype Asti Sorì. Left: field‐grown plant showing the characteristic green, elongated, and spineless capitulum cultivated in the Asti area (Piedmont, northwestern Italy). Right: longitudinal section of a representative capitulum highlighting the morphology of the receptacle and inner bracts.

Agronomic evaluation across two contrasting environments in the Asti area revealed trait‐specific differences but broadly overlapping phenotypic distributions between the tested sites. Plants cultivated at Santa Margherita (Costigliole d'Asti) showed greater variability in vegetative traits, particularly plant height and stem diameter, than those grown in Revigliasco (Figure 2). Yield‐related traits showed overlapping distributions between sites, while capitulum morphology traits, including head dimensions and shape ratio (L/H), showed broadly similar distributions. To quantify concordance between cultivation sites, Pearson correlation coefficients were calculated for the 12 shared line identifiers. The correlations were *r* = 0.376 (*p* = 0.229) for plant height, *r* = 0.550 (*p* = 0.064) for stem diameter, *r* = −0.107 (*p* = 0.784) for production, *r* = −0.058 (*p* = 0.881) for number of heads, *r* = 0.005 (*p* = 0.990) for mean head weight, *r* = 0.612 (*p* = 0.080) for head width, *r* = 0.070 (*p* = 0.858) for head height, and *r* = 0.750 (*p* = 0.020) for the shape ratio L/H. Production traits were evaluated using nine complete pairs (Table S2). Because each cultivation site was represented by a single field, these analyses describe site‐associated variation and do not constitute a replicated test of environmental effects. Overall, the observations provide descriptive support for using Asti Sorì as a focal material for genomic characterization, but do not by themselves demonstrate phenotypic stability, genomic distinctiveness, or molecular traceability across the entire ecotype.

**FIGURE 2:**
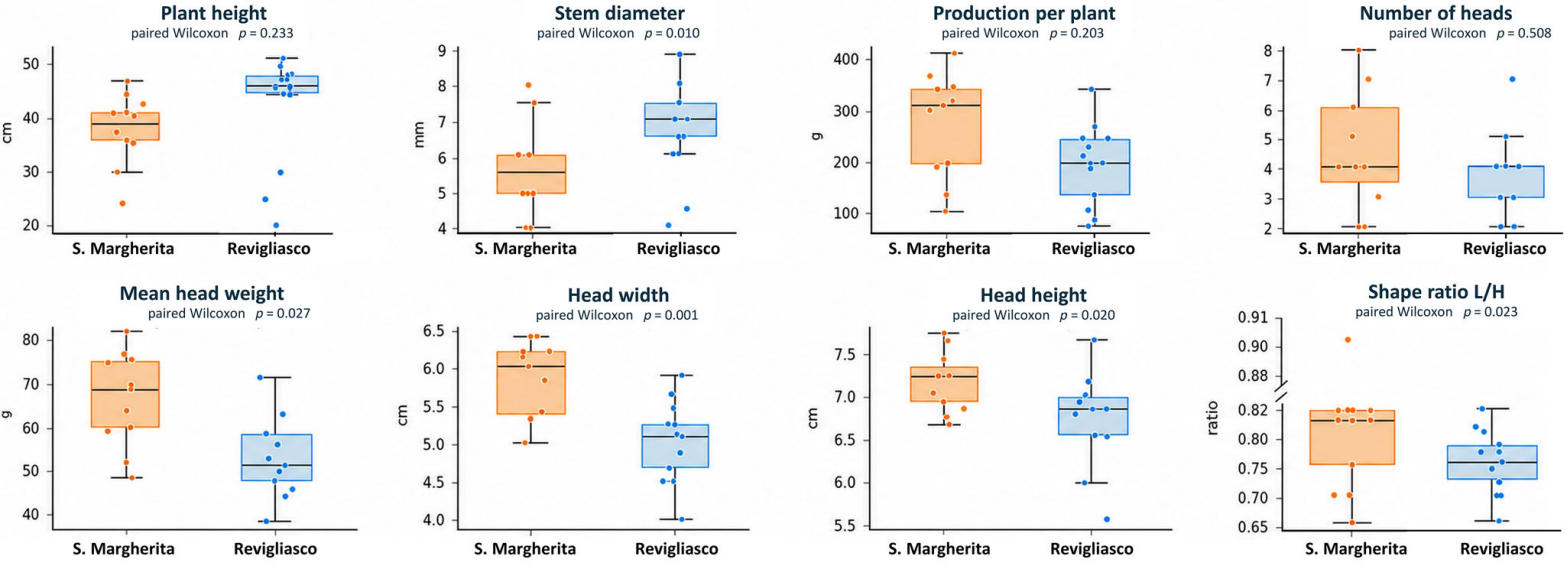
Phenotypic traits of the Asti Sorì ecotype evaluated at Santa Margherita (Costigliole d'Asti; orange) and Revigliasco (blue). Points represent individual line observations, boxplots summarize the site‐specific distributions, and exact paired Wilcoxon p‐values are reported above each panel. From top left to bottom right are reported: plant height; stem diameter; total production per plant; number of capitula per plant; mean head weight; capitulum width; capitulum height; capitulum shape ratio (L/H).

### 3.2 Reference‐guided genome reconstruction and annotation

A reference‐guided assembly approach enabled the generation of reconstructed genomes for all accessions, including the newly sequenced Asti Sorì, which were subsequently annotated using a combination of ab initio prediction and transcript‐supported methods. The resulting assemblies were produced after the RagTag reconstruction and annotated using a hybrid approach combining machine‐learning‐based gene prediction and transcriptomic evidence. Structural annotation identified between 34,074 genes in VT and 34,746 genes in C3 (Table 1). The combined use of Helixer and BRAKER3, supported by transcriptomic data (Scaglione et al., 2012), resulted in an expanded gene set compared to previous annotations (Acquadro et al., 2020). Overall, these reconstructed and annotated genomes provided the basis for subsequent comparative analyses and pangenome construction.

**TABLE 1:** Assembly and annotation statistics for the seven genome sequences included in the globe artichoke pangenome. The table reports the number of annotated protein‐coding genes, assembly size, number of scaffolds/sequences in the final RagTag output, N_50_, L_50_, GC content, and BUSCO completeness assessed at both genome and proteome levels. Scaffold/sequence counts include chromosome‐level pseudomolecules and unplaced sequences.

ID	Accession	Annotated genes	Assembly size (bp)	Final RagTag sequences	N_50_ (bp)	L_50_	BUSCO genome (%)	BUSCO proteome (%)	GC (%)
2C	Reference genome	34,018	727,464,971	18	44,809,927	7	97.6	95.2	31.832
A41	Cultivated cardoon	34,689	904,589,928	19	55,011,003	4	95.7	92.4	33.840
AS	Asti Sorì	34,225	954,584,786	19	58,355,108	3	95.2	92.1	33.052
C3	Romanesco C3	34,746	1,052,273,744	19	62,509,376	3	95.9	92.5	33.816
SP	Spinoso di Palermo	34,264	1,192,330,247	19	67,974,212	2	95.8	92.5	33.441
VS	Violetto di Sicilia	34,264	1,274,475,364	19	658,914,458	1	95.6	92.0	33.900
VT	Violetto di Toscana	34,074	1,144,616,130	19	65,685,680	2	95.7	92.4	33.466

### 3.3 Pangenome construction and architecture

To characterize genomic diversity in the analyzed *C. cardunculus* material, we constructed a pangenome by integrating the reconstructed genomes of five globe artichoke accessions and one cultivated cardoon accession with the globe artichoke reference genome. After redundancy removal and contaminant filtering, 131.3 Mb of non‐reference sequence was retained, representing sequence space not present in the reference assembly. RepeatMasker annotated approximately 106.2 Mb of the analyzed sequence, with the main classes corresponding to Unknown repeats (40.6 Mb), LTR/Copia (23.2 Mb), LTR/Gypsy (13.2 Mb), LINE/L2 (9.6 Mb), Simple_repeat (2.25 Mb), Low_complexity (0.175 Mb), and Satellite DNA (0.016 Mb). The complete repeat‐composition summary is provided in Table S3. Gene prediction on these retained non‐reference sequences identified 2894 non‐reference protein‐coding gene models. These were defined as non‐redundant protein‐coding models predicted on retained non‐reference pangenome sequences after redundancy removal and contaminant filtering. The designation “non‐reference” refers to their absence from the reference‐guided gene set used in this analysis and does not imply evolutionary novelty or absence from the species. Similarity‐based annotations and conserved‐domain support were evaluated separately. Among these, 1352 were supported by conserved domains (InterPro), and 1023 were assigned functional annotations including GO terms. The integration of these genes with those of the reference genome resulted in a pangenome comprising 37,198 protein‐coding genes and an assembled sequence span of approximately 0.86 Gb. The published genome‐size estimate of approximately 1.084 Gb refers to the expected haploid genome size, whereas the assembled 2C reference genome spans 727.5 Mb (Acquadro et al., 2020; Scaglione et al., 2016). The assembled pangenome spans approximately 858.8 Mb, corresponding to the 727.5‐Mb reference plus 131.3 Mb of non‐reference sequences. Thus, 0.86 Gb represents the assembled sequence content of the pangenome and is not intended to equal the estimated total genome size.

Based on gene presence across accessions, the pangenome was partitioned into core, softcore, shell, and cloud gene categories (Figure 3). A total of 30,817 genes were classified as core, being shared by all accessions, 1874 as softcore, shared by six accessions, 3471 as shell, shared by two to five accessions, and 580 as cloud genes, present in only one accession (Tables S4 and S5). The complete list of accession‐specific cloud genes and their functional annotation is provided in Table S6. Most genes were classified as core or softcore, indicating a high level of conservation across the analyzed germplasm, while a smaller fraction constituted the accessory genome, including shell and cloud components. Among the analyzed accessions, A41 showed the highest proportion of cloud genes (0.70%), whereas VT exhibited the lowest (0.07%; Table 2; Table S4; Figure 3).

**FIGURE 3:**
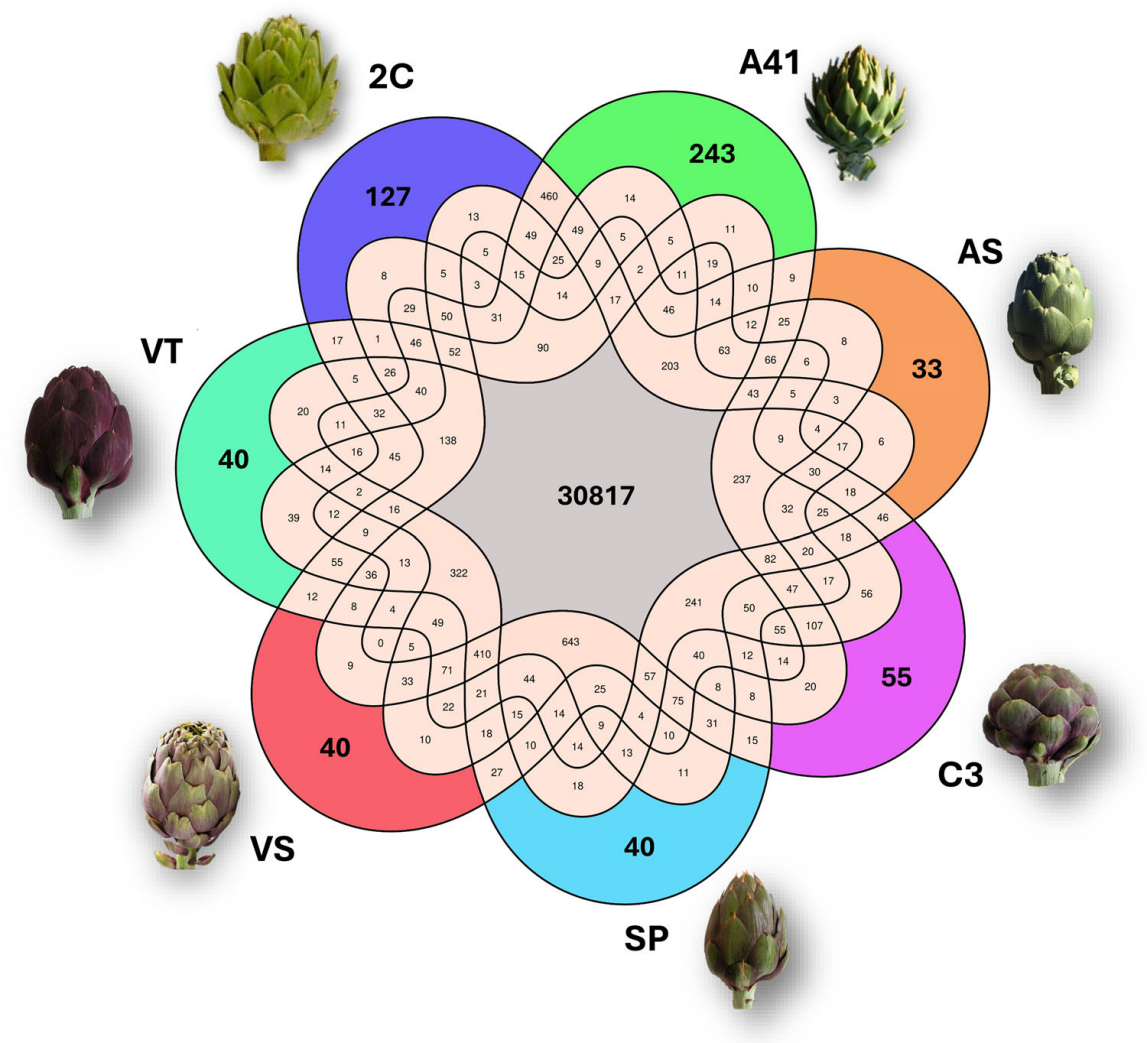
Gene presence/absence relationships among the seven genomes included in the globe artichoke pangenome. Numbers within the colored sectors indicate accession‐specific (cloud) genes, whereas overlapping regions correspond to genes shared by different combinations of accessions. Accessions included in the analyses were: Asti Sorì (AS), Romanesco C3 (C3), Violetto di Toscana (VT), Violetto di Sicilia (VS), Spinoso di Palermo (SP), cultivated cardoon Altilis 41 (A41), and the globe artichoke reference genotype 2C.

**TABLE 2:** Percentages for core, softcore, shell, and cloud are calculated among genes detected in each accession. “Absent (0/7)” genes are shown separately and are not counted as cloud genes.

Class	2C	A41	AS	C3	SP	VS	VT
Core	30,817 (90.59%)	30,817 (88.84%)	30,817 (90.04%)	30,817 (88.69%)	30,817 (89.94%)	30,817 (89.94%)	30,817 (90.44%)
Softcore	1231 (3.62%)	1552 (4.47%)	1736 (5.07%)	1784 (5.13%)	1671 (4.88%)	1637 (4.78%)	1633 (4.79%)
Shell	1843 (5.42%)	2077 (5.99%)	1639 (4.79%)	2090 (6.02%)	1736 (5.07%)	1754 (5.12%)	1599 (4.69%)
Cloud	127 (0.37%)	243 (0.70%)	33 (0.10%)	55 (0.16%)	40 (0.12%)	57 (0.17%)	25 (0.07%)
Absent (0/7)	3180	2509	2973	2452	2934	2933	3124

*Note*: Accession key: 2C, reference genome; A41, cultivated cardoon; AS, Asti Sorì; C3, Romanesco C3; SP, Spinoso di Palermo; VS, Violetto di Sicilia; VT, Violetto di Toscana.

The dynamics of pangenome expansion were assessed by modeling gene accumulation curves (Figure 4). The pangenome size increased with the addition of new genomes, reaching 37,198 genes with the inclusion of all seven genomes. The core genome, representing genes shared by all accessions, was estimated at 30,817 genes and decreased as additional genomes were incorporated, consistent with the expected behavior under Heaps’ law. Although the rate of new gene discovery decreased with each additional genome, indicating a tendency toward saturation within the sampled cultivated germplasm, the limited number of genomes included prevents definitive conclusions about the pangenome status of *C. cardunculus* across its full taxonomic and geographic range.

**FIGURE 4:**
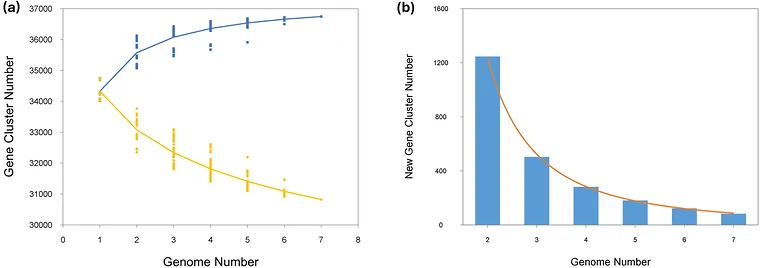
(a) Gene accumulation curves of the pangenome (blue) and the core genome (orange). These curves describe the number of new genes (pangenome) and genes in common (core genome) obtained by adding a new genome to a previous set. The blue vertical lines denote the pangenome size for each genome for comparison. The orange vertical lines show the core genome size for each genome for comparison. The curve is the least squares fit of the power law for the average values. (b) Curve (orange) for the number of new genes at each increase in the number of genomes.

Overall, the pangenome expansion curve showed a tendency toward saturation within the sampled cultivated germplasm, with a decreasing rate of additional gene‐model recovery as additional accessions were included. This pattern consisted of a conserved gene repertoire among the analyzed accessions, accompanied by a limited accessory component contributing to gene‐content variation.

To characterize the functional landscape of the globe artichoke pangenome, GO enrichment analysis was performed for each gene category using a custom *C. cardunculus* annotation database built from eggNOG‐mapper, InterProScan, and DIAMOND‐based similarity searches against Swiss‐Prot and NCBI RefSeq plant protein databases (see Materials and Methods). The pangenome displayed a marked functional stratification along the core‐to‐cloud axis (Figure 5). Transcriptional regulators and transporter‐encoding genes were significantly enriched in the core genome (BH‐corrected Fisher's test, *p* < 0.001), consistent with a conserved regulatory and metabolic backbone shared across all accessions. In contrast, genes involved in specialized (secondary) metabolism and defense responses showed a monotonically increasing enrichment from core to cloud categories, with the strongest signal observed for specialized metabolism (shell: *p* < 0.001; cloud: *p* < 0.01). Genes assigned to primary metabolism and housekeeping functions were uniformly distributed across all pangenome categories (*p* > 0.05), confirming that the observed gradient reflects genuine functional partitioning rather than annotation coverage biases (Table S7).

**FIGURE 5:**
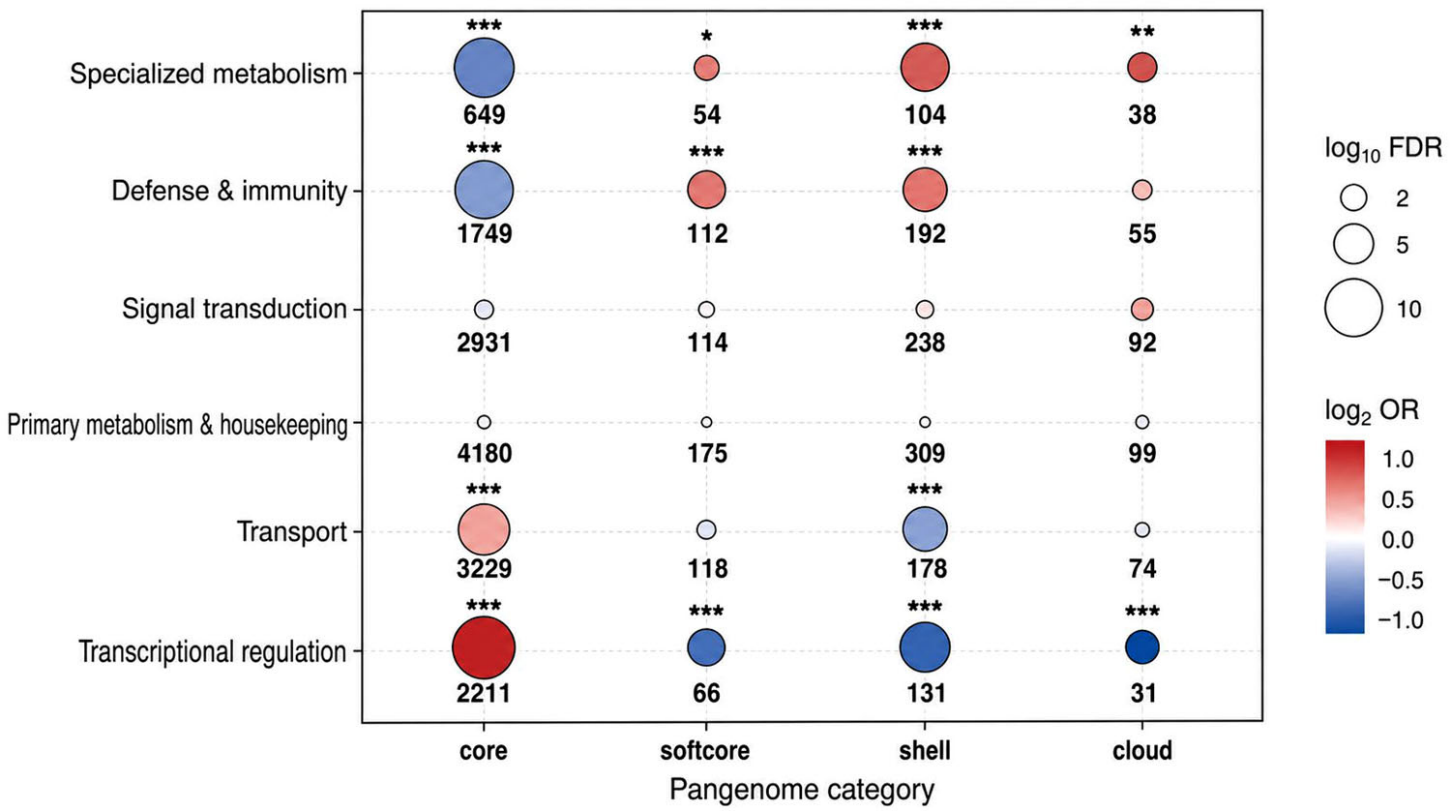
Functional stratification of the globe artichoke pangenome. Enrichment of curated functional categories along the core‐to‐cloud axis. Bubble size is proportional to enrichment significance (−log_10_ FDR), color indicates the enrichment odds ratio (log_2_ OR), and numbers below the bubbles indicate the number of genes contributing to each enriched functional category. Asterisks indicate statistically significant enrichments.

Of the 580 cloud genes, 397 were located on non‐reference contigs and 183 on reference‐derived contigs. RepeatMasker features overlapped 95 cloud genes, including 25 genes overlapping Low_complexity or Simple_repeat annotations. In addition, remapping of 217,883 originally unaligned sequence portions identified overlaps with 177 of the 580 cloud genes (30.5%), using ≥90% sequence identity, ≥80% query coverage, and MAPQ ≥ 20. These values provide a fragmentation/breakpoint proxy rather than an independent structural‐variant call (Table S8).

Detailed GO enrichment analysis of the accessory genome (shell and cloud genes combined; *n* = 4051) further resolved the biological functions associated with accession‐variable genes (Figure S1; Table S9). Within biological processes, isoprenoid and terpenoid biosynthetic processes were among the most significantly enriched terms (BH *p* < 0.01), consistent with the central role of sesquiterpene lactones and phenylpropanoids in globe artichoke quality traits and inter‐accession chemical diversity. Nitrate assimilation and nitric oxide biosynthesis were also enriched, pointing to variation in nitrogen metabolism and signaling across accessions. Among molecular function terms, transmembrane receptor tyrosine kinase activity and calcium channel activity showed the strongest enrichment, reflecting the diversity of immune receptor and signal transduction gene repertoires in the variable portion of the pangenome. Cellular component analysis revealed enrichment of photosynthetic membrane and plastid ribosomal subunit terms, indicating structural variation in plastid‐associated gene content (Figure S1).

Accession‐specific functional signatures were explored for the AS and the cultivated cardoon A41 accessions. Among the 33 genes uniquely present in Asti Sorì, GO enrichment analysis at a relaxed significance threshold (FDR < 0.10) identified terms associated with specialized metabolism, including sesquiterpene synthase activity (GO:0010334) and epoxide hydrolase activity, suggesting a distinctive secondary metabolite capacity in this ecotype. The annotations, predicted functions, homology evidence, and conserved‐domain assignments for these genes are reported in Table S6. However, the limited number of accession‐unique genes and the absence of significant enrichment at the conventional FDR < 0.05 threshold preclude firm conclusions. For the cultivated cardoon A41, functional annotation coverage was substantially reduced: 83% of the 243 A41‐unique genes corresponded to non‐reference pangenome gene models characterized by short, predicted protein sequences (median length 105 amino acids, compared with 310 amino acids for reference‐derived genes), a limitation inherent to de novo assembly‐based prediction. Only a single GO term reached significance for A41‐unique genes (oxidoreduction‐driven active transmembrane transporter activity; FDR = 0.004), precluding broader functional inference for the cardoon‐specific gene set.

### 3.4 Genome‐wide variation, accession relationships, and candidate diagnostic markers

Genome‐wide variant calling revealed extensive genetic variation across the analyzed accessions, with a predominance of heterozygous variants, consistent with the allogamous nature of *C. cardunculus*. The number of heterozygous variants differed among accessions, with the cultivated cardoon accession A41 showing the lowest number (approximately 2.9 million) and the Asti Sorì accession the highest (approximately 9.7 million; Figure 6).

**FIGURE 6:**
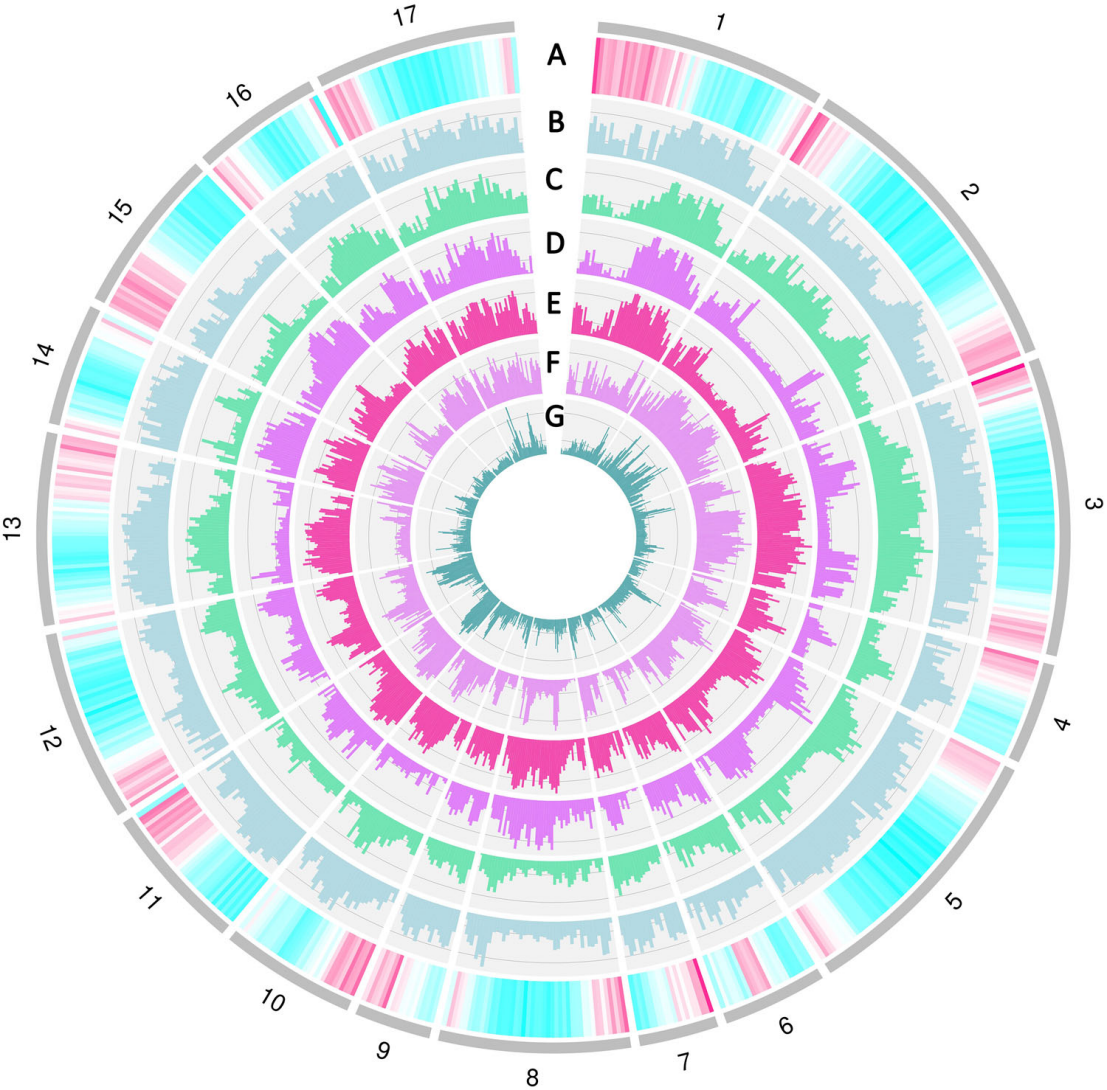
Circos diagram showing gene density in the reference genome (A) and the density of heterozygous single‐nucleotide polymorphisms (SNPs) in the Asti Sorì artichoke (B), Romanesco C3 (C), Violetto di Toscana (D), Violetto di Sicilia (E), Spinoso di Palermo (F), and the cultivated cardoon A41 (G).

Variant calling in AS identified 16,247,543 total variant records, including 14,891,580 SNPs and 1,355,963 INDELs. Heterozygous events accounted for 9,812,962 records (9,011,547 SNPs and 801,415 INDELs), whereas homozygous‐alternative events accounted for 6,434,581 records (5,880,033 SNPs and 554,548 INDELs) (Table S10). Genetic relationships among accessions were investigated using both SNP‐based and PAV‐based approaches derived from the pangenome. PCA based on genome‐wide SNPs revealed clear genetic differentiation among accessions (Figure S2A). PC1 and PC2 explained 29.35% and 17.22% of the total variance, respectively, corresponding to a cumulative variance of 46.57%. Because PCA components are descriptive ordination axes, no formal p‐value was assigned to individual components. The reference accession ‘2C’ and the cultivated cardoon A41 were clearly separated along PC1, indicating distinct genetic profiles. In contrast, VT, VS, and SP clustered closely together, suggesting a high degree of genetic similarity. The Asti Sorì accession occupied an intermediate position distinct from the other sampled accessions, whereas C3 showed greater divergence along PC2. This pattern is specific to the analyzed AS accession and should not be generalized to the entire Asti Sorì ecotype.

Maximum‐likelihood phylogenetic inference based on 34,583 pruned SNPs supported the clustering pattern observed in the PCA (Figure S2B). A41 and 2C formed a distinct clade, while SP, VS, and VT grouped together with high bootstrap support. Asti Sorì and C3 clustered separately, and a similar overall topology was observed using PAV‐based phylogenetic reconstruction, although differences in the relative positioning of some accessions were detected.

To complement the SNP‐ and PAV‐based analyses, candidate SVs were characterized across the six non‐reference accessions. After applying the predefined quality filters, 213,082 high‐confidence candidate SV records of at least 50 bp were retained. These included 102,943 deletions (DEL), 107,112 insertions (INS), 1536 duplications (DUP), 896 inversions (INV), and 595 translocations (TRA). The number of retained SV records ranged from 21,544 in A41 to 45,000 in AS (Table S11). A more stringent subset of large events, defined as non‐TRA variants with an absolute SVLEN of at least 1 kb together with all translocations, comprised 9099 records. These included 7533 deletions, 778 duplications, 193 inversions, and 595 translocations; no insertion ≥1 kb passed the filtering criteria. After clustering events with the same SV type and breakpoint coordinates within 100 bp and with a size difference not exceeding the larger of 100 bp or 20% of the event size, 5031 operationally non‐redundant SV clusters were identified. Of these, 1988 were shared by at least two accessions, whereas 3043 were observed in only one analyzed accession (Table S11). These results provide read‐supported evidence for multiple classes of candidate structural variation in the analyzed *C. cardunculus* material.

Taken together, these analyses indicate that both nucleotide variation and genome‐content variation contribute to the differentiation of globe artichoke accessions. SNP‐based analyses primarily captured overall genetic similarity, whereas PAV‐based analyses provided complementary information on accession‐level differences in gene content. The SV analysis further documented multiple classes of candidate structural variation relative to the composite pangenome reference.

### 3.5 Varietal relationships and reduced SNP fingerprinting using pangenome‐anchored GBS markers

Using the globe artichoke pangenome as a common coordinate framework, pangenome‐anchored GBS‐derived SNPs were analyzed across a panel of 45 accessions representative of the principal cultivated varietal groups. PCA based on the complete filtered SNP dataset revealed a clear genetic differentiation among varietal groups (Figure 7). PC1 and PC2 explained 24.59% and 12.14% of the total variance, respectively, corresponding to a cumulative variance of 36.73%. Eigenvalues and cumulative variance for all principal components are reported in Table S12. Because PCA components are descriptive ordination axes, no formal *p*‐value was assigned to individual components. The first principal component separated three major groups of accessions, with Spinosi materials positioned on the positive side of the axis, Catanesi and Violetti di Provenza accessions on the opposite side, and Romanesco, Violetto di Toscana, and related materials occupying intermediate positions. The Asti Sorì accession occupied a distinct position relative to the other groups; however, this pattern is specific to the analyzed accession and should not be interpreted as evidence of genomic distinctiveness of the entire Asti Sorì ecotype.

**FIGURE 7:**
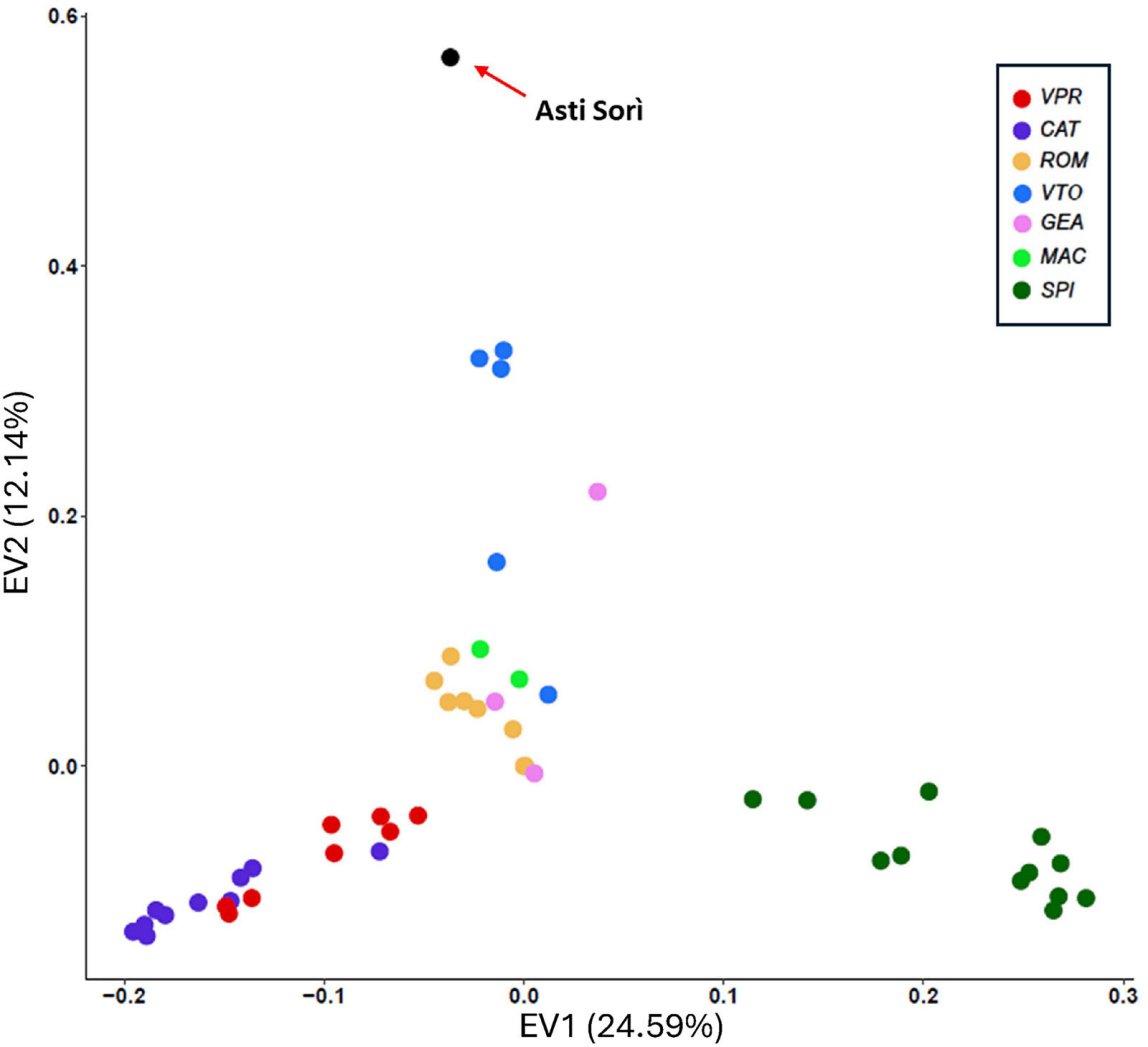
Principal component analysis (PCA) of the globe artichoke diversity panel based on the complete filtered set of pangenome‐anchored GBS (genotyping‐by‐sequencing)‐derived single‐nucleotide polymorphisms (SNPs). Accessions are colored according to the principal morpho‐agronomic typologies defined by Comino et al. (2016) and Rau et al. (2022): Catanesi (CAT), Violetti di Provenza (VPR), Violetti di Toscana (VTO), Romaneschi (ROM), Spinosi (SPI), Macau (MAC), and Green et al. (GEA). The Asti Sorì accession is indicated separately. PC1 (EV1) and PC2 (EV2) explained 24.59% and 12.14% of the total variance, respectively, corresponding to a cumulative variance of 36.71%. Eigenvalues, percentage of variance explained, and cumulative variance for all principal components, including PC3 and PC4, are reported in Table S12.

Population structure analysis resolved the analyzed accessions into distinct genetic groups broadly corresponding to the recognized morpho‐agronomic typologies of cultivated globe artichoke (Figure 8, right side). sNMF analysis identified *K* = 6 as the most informative subdivision level, consistent with the complex structure previously reported for globe artichoke germplasm. Catanesi and Violetti di Provenza accessions showed similar ancestry profiles with high ancestry coefficients, confirming their close genetic relationship. Most Romanesco accessions and all Violetto di Toscana accessions formed clearly differentiated groups, whereas Macau, Green et al. (GEA), and two Romanesco accessions displayed more admixed genetic backgrounds, occupying intermediate positions among the principal clusters. Spinosi accessions formed a distinct genetic group that was further subdivided into two major subclusters corresponding to their geographic origin, separating Sicilian spiny accessions from the Sardinian ones. The Asti Sorì accession showed a distinct and independent clustering profile relative to all other varietal groups, supporting the distinct genetic position of the analyzed Asti Sorì accession within the sampled germplasm.

**FIGURE 8:**
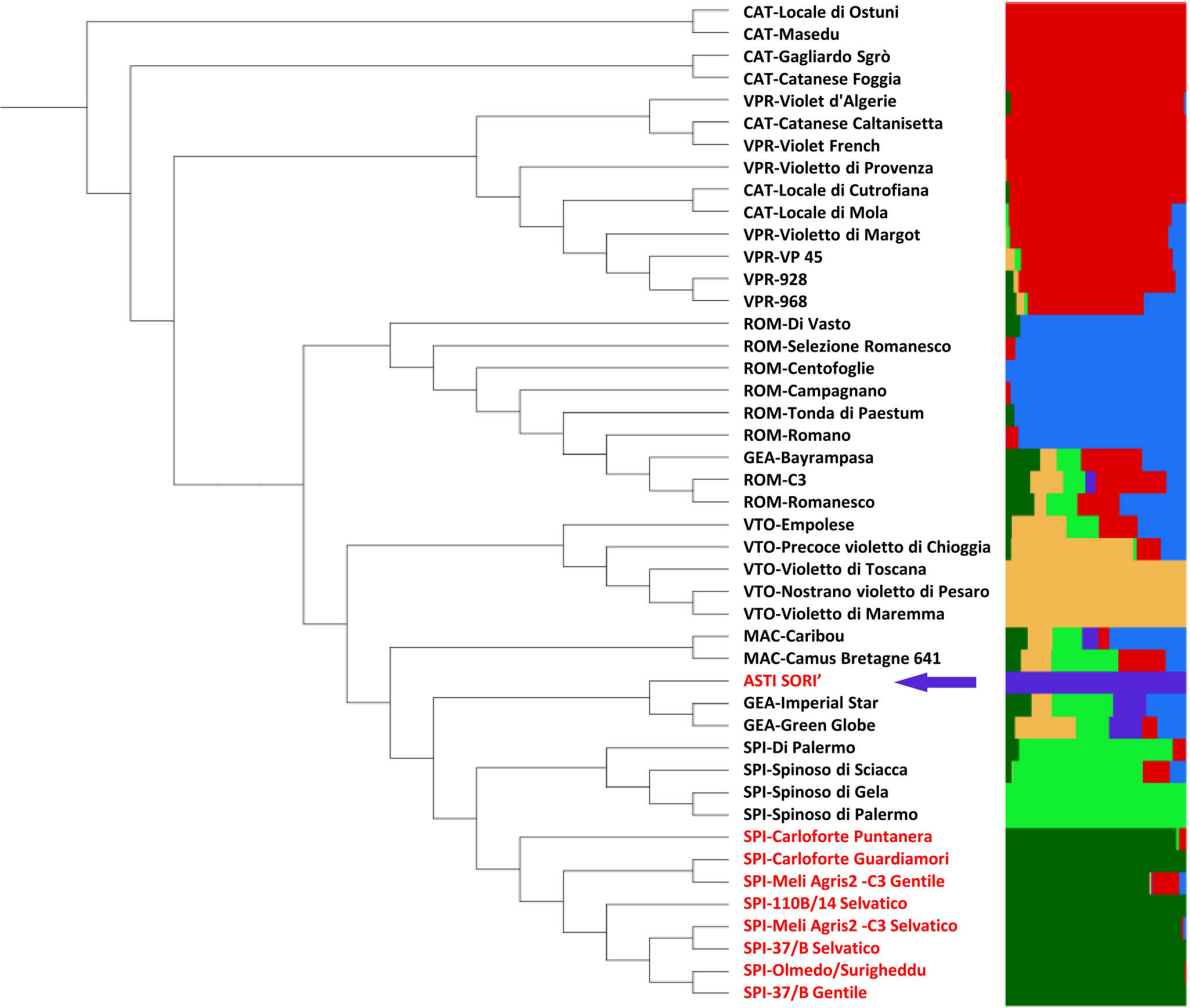
Population structure of the globe artichoke diversity panel based on pangenome‐anchored GBS (genotyping‐by‐sequencing)‐derived single‐nucleotide polymorphisms (SNPs). Right: sNMF ancestry coefficients for individual accessions at *K* = 6. Left: maximum‐likelihood phylogenetic tree inferred from the complete filtered SNP dataset. Accessions are labeled according to the principal morpho‐agronomic typologies as in Figure 7. Sardinian accessions are highlighted in red within the SPI group; Asti Sorì accession is indicated separately.

Maximum‐likelihood phylogenetic reconstruction broadly supported the clustering patterns observed in both the PCA and sNMF‐based population structure analysis (Figure 8, left side). Catanesi and Violetti di Provenza accessions consistently clustered together while maintaining detectable internal differentiation. Romanesco accessions formed a coherent phylogenetic group, including the two accessions that displayed partially admixed ancestry profiles in the sNMF analysis. Spinosi accessions clustered into a distinct and well‐supported clade, within which the subdivision between Sicilian and Sardinian materials was maintained, confirming the geographic structure previously highlighted by the sNMF analysis. The Asti Sorì accession grouped within the broader cluster of green‐headed accessions, consistent with its morphological characterization as a green, spineless varietal type, while maintaining a distinct phylogenetic position relative to the remaining green‐headed materials.

To evaluate the feasibility of reduced SNP‐based varietal fingerprinting, iterative random subsampling analyses were performed on the complete pangenome‐anchored SNP dataset. For panel sizes of 50, 75, and 100 SNPs, 100 random replicates were evaluated by Pearson correlation between the pairwise genetic distance matrix derived from each candidate panel and that obtained from the complete SNP dataset (Table S13). The best‐performing 50‐SNP panel (Pearson's *r *= 0.96; Tables S13 and S14) was retained for evaluation in the final 45‐accession germplasm panel. Using the final 45‐accession panel, with missing genotypes retained as explicit missing states in the profile encoding, random 50‐SNP panels produced 44.93 ± 0.26 distinct profiles (range 44–45; 100 iterations), whereas the selected 50‐SNP panel produced 44 distinct observed profiles among the 45 accessions. These analyses represent an internal resampling assessment rather than independent validation because optimization and evaluation used the same germplasm collection. The 50‐SNP panel should therefore be considered a preliminary candidate tool requiring validation in independent germplasm collections. Phylogenetic reconstruction based exclusively on this 50‐SNP subset largely recapitulated the principal varietal structure observed with the full dataset. Although some internal relationships among closely related accessions differed from those inferred using the complete SNP matrix, all major varietal groups remained recognizable. In particular, the Asti Sorì accession maintained a clearly differentiated phylogenetic position, supporting the potential utility of the reduced marker set for varietal discrimination and molecular fingerprinting within the analyzed germplasm (Figure 9).

**FIGURE 9:**
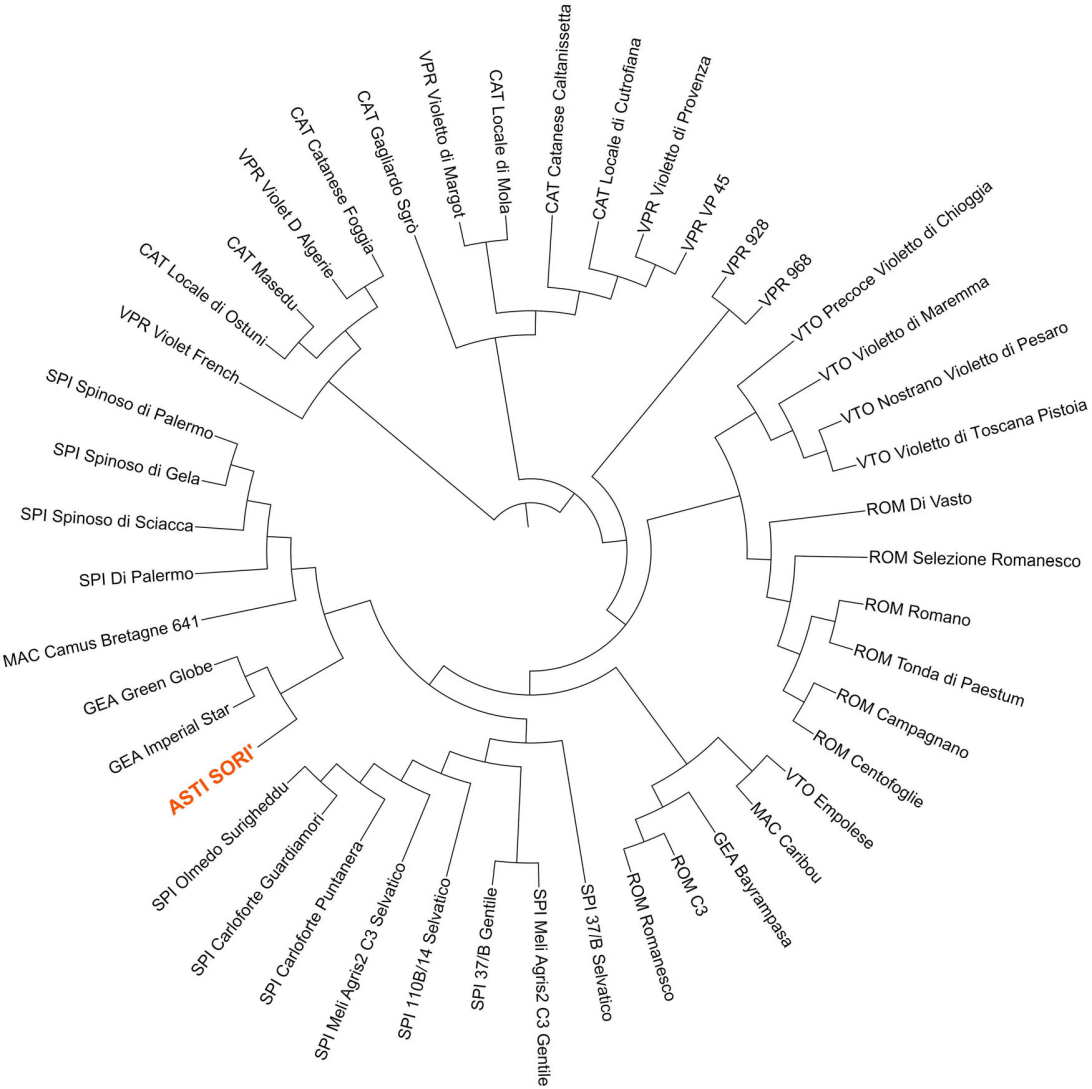
Radial phylogenetic tree inferred from the reduced set of 50 pangenome‐anchored single‐nucleotide polymorphisms (SNPs) selected for varietal fingerprinting. Accessions are labeled according to the principal morpho‐agronomic typologies reported in Figure 7; Asti Sorì accession is indicated in red.

## 4 DISCUSSION

We developed the first pangenomic framework for globe artichoke and explored SNP‐based approaches for varietal discrimination. The globe artichoke pangenome revealed a genomic architecture dominated by a large, conserved core gene set, complemented by a smaller accessory component comprising genes variably distributed across accessions. This structure is broadly consistent with pangenomes reported in other crop species, where core gene content generally represents conserved developmental and metabolic functions, whereas accessory genes are often enriched for functions related to environmental response, secondary metabolism, disease resistance, and transposable element activity (Bayer et al., 2020; Bozan et al., 2023; Gaccione et al., 2025; Golicz et al., 2015). The pangenome accumulation curves suggested a tendency toward saturation within the sampled cultivated germplasm. However, the limited number of genomes analyzed, all of cultivated origin, prevents strong conclusions about the overall openness of the *C. cardunculus* pangenome across its full taxonomic and geographic range. Inclusion of wild cardoon (*C. cardunculus* var. *sylvestris*) and more cultivated cardoon (*C. cardunculus* var. *altilis*) accessions in future pangenome projects will be important to capture the full extent of structural and sequence variation within this species complex. This first pangenomic framework therefore provides not only a genomic resource for globe artichoke, but also a baseline for distinguishing conserved species‐level features from accession‐variable genomic content.

The biological relevance of this pangenomic resource became evident when gene‐content variation was interpreted in functional terms, revealing a clear functional stratification across pangenome categories. Transcriptional regulators and transporter genes were consistently enriched in the core genome, suggesting that conserved regulatory and transport‐related functions represent part of the shared genomic infrastructure across the analyzed accessions. In contrast, the accessory genome showed enrichment for specialized metabolism, particularly isoprenoid and terpenoid biosynthetic processes, together with defense‐related functions including receptor kinase signaling and calcium channel activity. This distribution suggests that genes variably present across accessions are not exclusively residual or annotation‐derived elements, but include functional categories potentially associated with adaptive and quality‐related traits. A particularly relevant result was the increasing enrichment of specialized metabolism from softcore to cloud categories. This trend suggests that genes located at the periphery of the pangenome may preferentially contribute to accession‐specific chemical diversity. Similar patterns have been reported in other crop pangenomes, including tomato and maize, where dispensable genome content is disproportionately associated with adaptive traits, disease resistance, and secondary metabolite diversification (Gao et al., 2019; Hirsch et al., 2014; Shi et al., 2026; S. Yang et al., 2025; Zhou et al., 2022). In globe artichoke, this observation is especially relevant because several secondary metabolites, including sesquiterpene lactones such as cynaropicrin and phenolic compounds such as chlorogenic acids, are central to product quality, nutritional value, and cultivar differentiation. The enrichment of terpenoid‐ and phenylpropanoid‐related functions in the accessory genome therefore suggests that comparative pangenomics may provide a useful framework for prioritizing candidate genes contributing to chemical and adaptive diversity in *C. cardunculus*. However, because the pangenome was constructed from only seven genome sequences, the shell and cloud assignments should be considered sampling‐dependent operational categories rather than definitive species‐wide classes. The addition of further accessions could reassign genes between these categories, particularly between shell and cloud.

Population structure analyses based on genome‐wide SNPs and PAV revealed broadly consistent, but not fully overlapping, patterns of accession relationships. This partial divergence is expected because SNP‐based analyses capture nucleotide‐level variation distributed across the genome, whereas PAV‐based analyses describe differences in gene content. These two layers therefore provide complementary rather than redundant views of diversity, as also observed in other crops where structural and nucleotide variation can reflect partially independent evolutionary histories (Jayakodi et al., 2024; Qin et al., 2021; Zhou et al., 2022). Both approaches nevertheless consistently identified Asti Sorì as distinct from the other resequenced accessions, reinforcing its genomic individuality within the analyzed material.

The additional structural‐variant analysis complemented the SNP‐ and PAV‐based characterization by revealing a further layer of genome variation among the analyzed accessions. After stringent quality filtering, 213,082 high‐confidence candidate SV records of at least 50 bp were identified, including deletions, insertions, duplications, inversions, and translocations. The more conservative subset of large events comprised 9099 records, which were consolidated into 5031 operationally non‐redundant clusters; 1988 clusters were shared by at least two accessions, whereas 3043 were private to a single accession. These results suggest that structural variation may contribute to accession‐level genomic differentiation beyond SNP and gene‐content variation. However, the calls are relative to the composite pangenome reference, which contains sequence derived from the analyzed accessions, and should therefore be interpreted as read‐supported candidate SVs rather than as an unbiased, species‐wide catalogue. In this view, duplication calls represent candidate copy‐number‐related events and not independently validated CNVs.

The high heterozygosity observed across the resequenced accessions, particularly in Asti Sorì, is consistent with the allogamous reproductive biology of globe artichoke and with the complex allelic landscape expected in clonally maintained, outbreeding varieties. In such crops, vegetative propagation can maintain highly heterozygous genotypes over time, while local selection and farmer‐mediated clonal maintenance may preserve combinations of alleles associated with agronomic performance or local adaptation. From this perspective, the genomic distinctiveness of Asti Sorì should not be interpreted as evidence that the analyzed accession exhaustively represents the ecotype, but rather as evidence that the local material contains a recognizable genomic component that can be targeted for further characterization.

These observations raised a practical question: whether the genomic variation detected in the pangenome could be translated into robust varietal discrimination tools. The broader GBS panel comprised a globe artichoke germplasm collection representing the major cultivated varietal groups (Comino et al., 2016; Rau et al., 2022). This resource provides an appropriate population‐level context in which to test whether variants identified from resequencing data can contribute to varietal discrimination. The identification of accession‐specific variants from the resequencing panel represented a first route toward candidate diagnostic markers for the other analyzed accessions as well as for Asti Sorì. However, variants that appear private within a small discovery panel may not retain diagnostic value when broader germplasm diversity is considered. This is a general limitation of accession‐specific marker discovery based on restricted genome panels and highlights the need to evaluate marker informativeness at the population scale. However, the 100 randomizations represented an internal resampling assessment rather than independent validation, and the optimized panel was evaluated using the same germplasm collection used for its development. The 50‐SNP panel should therefore be considered a preliminary candidate tool requiring validation in independent germplasm collections and across technical genotyping platforms.

For this reason, the study moved from private‐variant discovery to a population‐level SNP fingerprinting strategy using the 45‐accession GBS dataset. Genome‐wide GBS‐derived SNPs resolved the main varietal groups with high confidence, and a reduced set of 50 markers was sufficient to recapitulate the major axes of genetic differentiation observed in the full dataset. This result indicates that, within the analyzed germplasm, a compact set of pangenome‐anchored SNPs can capture the principal structure of cultivated globe artichoke diversity. The 50‐SNP panel identified here therefore provides a computationally validated candidate resource for varietal fingerprinting in globe artichoke. The potential applications of this candidate marker set include support for DUS testing, product traceability, and conservation monitoring of traditional ecotypes. In DUS‐oriented contexts, standardized molecular profiles may complement morphological descriptors, particularly for clonally propagated crops in which phenotypic evaluation can be influenced by environment and management conditions (C. J. Yang et al., 2021). For traceability, low‐cost genotyping assays could support the verification of varietal origin along production chains. For conservation, molecular fingerprinting may help monitor the identity and stability of local ecotypes such as Asti Sorì, for which standardized genomic tools are currently lacking. In this sense, the integration of pangenome information with reduced SNP panels provides a practical framework for translating comparative genomics into applied varietal identification.

The 50‐SNP panel should be considered a candidate resource rather than a ready‐to‐use diagnostic tool. Conversion of the selected SNPs into deployable genotyping assays, such as KASP probes or targeted amplicon sequencing, will require additional technical development and validation. More importantly, the discriminatory performance of the panel should be evaluated on independent germplasm collections before routine diagnostic use can be proposed. The 45‐accession GBS panel analyzed here, while representative of major Italian varietal groups, does not capture the full diversity of globe artichoke preserved in national and international germplasm repositories. In particular, the ability of the marker set to resolve closely related accessions within the same varietal group, a critical requirement for DUS‐oriented applications, remains to be established.

Local ecotypes of crop species may harbor allele combinations shaped by long‐term farmer selection under specific environmental and agronomic contexts, and their genomic characterization represents an important step toward molecular traceability, conservation, and formal recognition (Lazaridi et al., 2024; Ramirez‐Villegas et al., 2022). In the case of Asti Sorì, field evaluation across two cultivation sites in the Asti area supported the agronomic consistency of the local material. This consistency is compatible with the clonal propagation of globe artichoke and supports the relevance of the ecotype as a defined target for molecular characterization, while not implying that a single accession fully captures its intra‐ecotype diversity. Although only one representative Asti Sorì accession was used for whole‐genome resequencing and pangenome construction, this accession should be interpreted as a genomic entry point into the ecotype rather than as a complete description of its genetic breadth. Notably, Asti Sorì was consistently resolved as a distinct accession in both whole‐genome sequencing‐based and GBS‐based population analyses, supporting its individuality within the broader landscape of Italian globe artichoke diversity. In this framework, the fingerprinting analysis shows that Asti Sorì is distinguishable from the other analyzed materials, the pangenome indicates the presence of accession‐specific gene‐content features, while the reduced SNP panel provides a concrete route to monitor, protect, and valorize this local ecotype. These results highlight the value of local ecotypes as reservoirs of unique diversity and as useful case studies for developing pangenome‐enabled fingerprinting approaches applicable to varietal protection, conservation, and breeding.

Overall, this study provides a first pangenomic resource for globe artichoke and demonstrates how local ecotypes can serve as informative entry points for integrating comparative genomics, population structure analysis, and molecular fingerprinting. The results support the genomic distinctiveness of Asti Sorì within the analyzed material, reveal a pangenome organization dominated by conserved gene content but enriched for functionally relevant accessory components, and identify a compact set of candidate SNPs for varietal discrimination.

Future research should expand the pangenome to include additional cultivated globe artichoke, cultivated cardoon, and wild cardoon accessions, as well as independent clones of the Asti Sorì ecotype. Long‐read and Hi‐C sequencing would improve assembly contiguity and enable more comprehensive characterization of SVs, including inversions, translocations, and copy‐number variation. Functional validation of accession‐variable genes, particularly those associated with specialized metabolism and defense responses, will also be required. Finally, the candidate 50‐SNP panel should be evaluated across independent germplasm collections and technical platforms and converted into accessible genotyping assays, such as KASP or targeted amplicon markers, for practical varietal identification and molecular traceability. Such developments will be essential to transform the resources generated here into robust tools for varietal protection, traceability, conservation, and breeding in *C. cardunculus*.

## 5 CONCLUSIONS

This study developed a first pangenomic framework for globe artichoke, using the Italian local ecotype Asti Sorì as an entry point. The pangenome captured a conserved core gene repertoire alongside an accessory component reflecting accession‐specific content, with accumulation curves suggesting a tendency toward saturation within the sampled cultivated germplasm. SNP and PAV analyses provided complementary and broadly consistent views of genomic diversity, consistently resolving Asti Sorì as a genomically distinct accession within the analyzed collection. simple sequence repeat loci and accession‐specific private variants, while informative in a limited‐panel context, proved insufficient or uncertain as robust diagnostic tools for broader varietal discrimination. GBS‐derived SNPs resolved well‐supported varietal groups across 45 accessions, and a reduced panel of 50 SNPs recapitulated the main population structure of the full dataset within the analyzed germplasm. This compact marker set provides a practical candidate resource for varietal fingerprinting, DUS testing, and the molecular protection of traditional Italian globe artichoke ecotypes. Further validation across independent germplasm collections will be required to establish this panel as a standardized diagnostic assay.

## AUTHOR CONTRIBUTIONS

**Ezio Portis**: Conceptualization; data curation; formal analysis; funding acquisition; investigation; methodology; resources; supervision; validation; visualization; writing—original draft; writing—review and editing. **Edoardo Vergnano**: Data curation; formal analysis; investigation; software; visualization; writing—original draft; writing—review and editing. **Luciana Gaccione**: Data curation; formal analysis; software; writing—original draft; writing—review and editing. **Alberto Acquadro**: Conceptualization; investigation; methodology; supervision; visualization; writing—original draft; writing—review and editing. **Cinzia Comino**: Conceptualization; investigation; methodology; supervision; visualization; writing—original draft; writing—review and editing. **Cristiano Carli**: Funding acquisition; resources; supervision; writing—original draft; writing—review and editing. **Lorenzo Barchi**: Conceptualization; formal analysis; investigation; methodology; software; supervision; visualization; writing—original draft; writing—review and editing. **Matteo Martina**: Conceptualization; data curation; formal analysis; investigation; methodology; software; supervision; visualization; writing—original draft; writing—review and editing.

## CONFLICT OF INTEREST STATEMENT

The authors declare no conflict of interest.

## Supporting information




**Supplementary Figure 1**. GO enrichment of accessory genome genes. Top enriched BP, MF, and CC terms; bar length indicates gene count and color indicates FDR.

**Supplementary Figure 2**. Genome‐wide relationships among the analyzed globe artichoke accessions based on SNP and presence/absence variation (PAV) data. (A) Principal component analysis (PCA) performed using 34,583 pruned SNPs derived from the globe artichoke pangenome. (B) Maximum‐likelihood phylogenetic trees inferred from SNP data (top) and presence/absence variation (PAV) data (bottom). Accessions included in the analyses were: ‘Asti Sorì’ (AS), ‘Romanesco C3’ (C3), ‘Violetto di Toscana’ (VT), ‘Violetto di Sicilia’ (VS), ‘Spinoso di Palermo’ (SP), cultivated cardoon ‘Altilis 41’ (A41), and the globe artichoke reference genotype ‘2C’.

**Table S1**. Globe artichoke accessions included in the GBS‐based diversity panel used for population structure analysis and reduced SNP fingerprinting. For each accession or clone, the corresponding morpho‐phenotypic typology is reported according to Comino et al. (2016) and Rau et al. (2022). Groups include “Catanesi” (CAT), “Violetti di Provenza” (VPR), “Violetti di Toscana” (VTO), “Romaneschi” (ROM), “Macau” (MAC), “Spinosi” (SPI), and “Green et al.” (GEA).
**Table S2**. Cross‐site phenotypic correlations for ‘Asti Sorì’. Pearson correlations between the two cultivation sites for shared line identifiers. Production traits were evaluated using nine complete pairs, whereas the remaining traits were evaluated using 12 pairs.
**Table S3**. Repeat composition of retained non‐reference sequence. RepeatMasker summary for the 131,316,682 bp of retained non‐reference sequence.
**Table S4**. Pangenome gene‐category classification. For each pangenome gene, the number of accessions in which it was detected as present (n_present) and the corresponding operational category are reported. Genes with n_present = 0 are retained as a separate absent class.
**Table S5**. Full gene presence/absence matrix. Complete presence/absence matrix for the seven pangenome accessions, including core, softcore, shell, cloud, and absent calls. The compact gene‐category classification is provided separately in Table S4.
**Table S6**. Accession‐specific cloud genes and functional annotation. Cloud genes private to each of the seven pangenome accessions are reported together with their transcript and gene identifiers, predicted products, Gene Ontology terms, and database cross‐references.
**Table S7**. Functional enrichment across pangenome macro‐categories. Enrichment statistics used for the functional‐stratification analysis, including odds ratios, log_2_‐transformed odds ratios, and Benjamini–Hochberg‐adjusted significance values.
**Table S8**. Cloud‐gene overlap with repeat, low‐complexity, and breakpoint‐proxy features. Summary of sequence‐context overlaps used to assess whether cloud genes may be associated with repetitive or potentially fragmented assembly regions.
**Table S9**. GO enrichment results. Gene Ontology enrichment results for the accessory genome, accession‐specific gene sets, and pangenome macro‐categories are reported. The table includes the number and identifiers of genes contributing to each enriched term, together with GeneRatio, BgRatio, odds ratios, and adjusted significance values.
**Table S10**. Variant composition in the ‘Asti Sorì’ accession. Breakdown of total variant records by variant class and genotype category, including SNPs, INDELs, heterozygous variants, and homozygous‐alternative variants.
**Table S11**. Candidate structural‐variant summary. Read‐supported candidate SV records called with Dysgu against the composite pangenome reference and retained after the predefined quality filters. The table summarizes SV classes, large‐event subsets, and operationally non‐redundant SV clusters.
**Table S12**. PCA eigenvalues, percentage of variance explained, and cumulative variance for all 44 principal components calculated from the filtered 45‐sample GBS panel based on 291 autosomal SNPs.
**Table S13**. Performance of random SNP subsampling panels. Summary statistics across 100 random replicates for each tested panel size, measured by Pearson correlation with the pairwise genetic‐distance matrix derived from the complete GBS dataset.
**Table S14**. Reduced SNP panels selected for varietal fingerprinting. Genomic coordinates and reference/alternative alleles of the 50‐, 75‐, and 100‐marker panels identified through random subsampling.

## Data Availability

The plant material analyzed in this study consisted of vegetatively propagated clonal accessions corresponding to the germplasm sources described by Acquadro et al. (2017), Rau et al. (2022), and Martina et al. (2024). As these materials are maintained as clonal accessions rather than publicly distributed seed accessions, no PI numbers are available. Requests for living material should be addressed to the corresponding authors or curators of the original collections, subject to material availability and applicable material‐transfer requirements. The globe artichoke pangenome assembly, the seven accession‐level genome assemblies, the corresponding GFF3 annotations, and the predicted protein sequences have been deposited in Zenodo and are available at https://doi.org/10.5281/zenodo.22305104. Raw sequencing reads are available under NCBI BioProject PRJNA1481990.
